# Supplementary material for: Non-Structural Proteins (Nsp): A Marker for Detection of Human Coronavirus Families
Source: Pathogens. 2023 Sep 21;12(9):1185. doi: 10.3390/pathogens12091185 (PMC10537875; doi:10.3390/pathogens12091185)
Supplement: Supplementary file 1 [file pathogens-12-01185-s001.zip › pathogens-2558046-supplementary.pdf]

SUPLEMENTARY INFORMATION

Table S1. *Main types of coronaviruses that infect humans.*

Type coronavirus	Accesion	Description/genome	Length genoma (nt)	Protein	Host	1ab polyprotein/ ORF1ab protein
		Alphacoronavirus				
HCoVs-229E	NC_002645	Human coronavirus 229E	27317 nt	8	Human, vertebrates strain: 229E	NP_073549.1
HCoVs-NL63	MG772808.1	Human coronavirus NL63	27553 nt	6	Human strain: Amsterdam I	AVA2672.1
		Betacoronavirus				
HCoVs-OC43	NC_006213	Human coronavirus OC43	30741 nt	10	Human, vertebrates strain: ATCC VR-759; serotype: OC43	YP_009555238.1
HCoVs-HKU1	NC_006577	Human coronavirus HKU1	29926 nt	9	Human isolate: HKU1	YP_173236.1
MERS-CoV	NC_19843	Middle East respiratory syndrome-related coronavirus	30119 nt	11	Human, vertebrates strain: HCoV-EMC; isolate: HCoV-EMC/2012	YP_009047202.1
MERS-CoV-Eng	NC_038294	Middle East respiratory syndrome-related coronavirus, England	30111 nt	11	Human, vertebrates strain: England 1; isolate: H123990006	YP_007188577.3
SARS-CoV	KY352407.1[a]	Severe acute respiratory syndrome-related coronavirus	29274 bp	10	Human, vertebrates Strain: BtKY72	APO40578.1

SARS-CoV-2	NC_045512	Severe acute respiratory syndrome-related coronavirus 2	29903 nt	12	Human, vertebrates isolate: Wuhan-Hu-1	YP_009724389.1
SARS-CoV-Tor2	NC_04718	Severe acute respiratory syndrome-related coronavirus Tor2	29751 nt	15	Human Isolate: patient #2 with severe acute respiratory syndrome (SARS)	NP_828849.7
		Gammacoronavirus[b]				QDY92334.1
AcCoV-Duck	NC_048214	Duck avian coronavirus	27754 nt	12	Human, vertebrates isolate:DK/GD/27/2014	YP_009825006.1
AcCoV- Turkey	NC_010800	Turkey avian coronavirus	27657 nt	11	Human, vertebrates isolate: MG10	YP_001941164.2
		Deltacoronavirus[c]				
Porcine coronavirus	MW685622.1	Porcine coronavirus HKU15	25444	1	Porcine Isolate: PDCoV/Haiti/Human/0081-4/2014	QWE80491.1
Sparrow deltacoronavirus	MG812375	Sparrow deltacoronavirus HKU17	25795 nt	9	Passeridae Isolate PDCoV/Haiti/Human/0081-4/2014	AWV67106.1

a *Complete genomic sequence of BtKY72, which is closely related to BtCoV/BM48-31/ Bulgaria/2008, a severe acute respiratory syndrome (SARS)-related virus from Rhinolophus bats in Europe.*

b, c: *Group of coronaviruses with a route of transmission through birds and porcine.*

Table S2. *Proportion of homologous families of alpha, beta, gamma and delta coronaviruses.*

Genome	Genes	Genes Homologs	Genes Singletons	Homolog Families
Alphacoronavirus				
HCoVs-229E (NC_002645)	7	2	5	2
HCoVs-NL63 (MG772808.1)	6	2	4	2
Betacoronavirus				
HCoVs-OC43 (NC_006213)	7	5	2	5
HCoVs-HKU1 (NC_006577)	8	5	3	5
MERS-CoV (NC_19843)	10	8	2	8
MERS-CoV-Eng (NC_038294)	9	9	0	9
SARS-CoV (KY352407.1)	10	7	3	7
SARS-CoV-2 (NC_045512)	11	8	3	8
SARS-CoV-Tor2 (NC_04718)	13	8	5	8
Gammacoronavirus				
AcCoV-Duck (NC_048214)	12	7	5	6
AcCoV-Turkey (NC_010800)	11	7	4	6
Deltacoronavirus				
Porcine HKU15 (MW685622.1)	7	7	0	7
Sparrow HKU17 (MG812375)	9	7	2	7

**Functional families were calculated with Build Pangenome with OrthoMCL (KBaseGenomes).*

Table S3. *Comparison families of alpha, beta, gamma and delta coronaviruses genomes*

**Genome**	**Families /Functions***												
	AcCoV-Turkey	AcCoV-Duck	Sparrow HKU17	Porcine HKU15	MERS-CoV-Eng	HCoVs-229E	SARS-CoV	HCoVs-HKU1	HCoVs-NL63	HCoVs-OC43	MERS-CoV	SARS-CoV-Tor2	SARS-CoV-2
AcCoV-Turkey	10<br>11	6<br>10	2<br>3	2<br>3	1<br>0	2<br>3	1<br>0	1<br>2	2<br>4	1<br>3	0<br>4	0<br>2	0<br>2
AcCoV-Duck	6<br>10	11<br>11	2<br>2	2<br>2	1<br>0	2<br>2	1<br>0	1<br>1	2<br>3	1<br>2	0<br>3	0<br>2	0<br>1
Sparrow HKU17	2<br>3	2<br>2	9<br>9	7<br>6	1<br>0	2<br>3	1<br>0	1<br>2	2<br>3	1<br>3	0<br>3	0<br>2	0<br>1
Porcine HKU15	2<br>3	2<br>2	7<br>6	7<br>7	1<br>0	2<br>3	1<br>0	1<br>2	2<br>3	1<br>3	0<br>3	0<br>2	0<br>1
MERS-CoV-Eng	1<br>0	1<br>0	1<br>0	1<br>0	9<br>1	1<br>0	2<br>1	1<br>0	1<br>0	1<br>0	8<br>0	1<br>0	1<br>0
HCoVs-229E	2<br>3	2<br>2	2<br>3	2<br>3	1<br>0	7<br>8	1<br>0	1<br>1	2<br>4	1<br>3	0<br>3	0<br>1	0<br>2
SARS-CoV	1<br>0	1<br>0	1<br>0	1<br>0	2<br>1	1<br>0	10<br>1	1<br>0	1<br>0	1<br>0	1<br>0	6<br>0	6<br>0
HCoVs-HKU1	1<br>2	1<br>1	1<br>2	1<br>2	1<br>0	1<br>1	1<br>0	8<br>9	1<br>1	5<br>1	0<br>1	0<br>3	0<br>5
HCoVs-NL63	2<br>4	2<br>3	2<br>3	2<br>3	1<br>0	2<br>4	1<br>0	1<br>1	6<br>6	1<br>3	0<br>4	0<br>1	0<br>1
HCoVs-OC43	1<br>3	1<br>2	1<br>3	1<br>3	1<br>0	1<br>3	1<br>0	5<br>1	1<br>3	8<br>8	0<br>3	0<br>1	0<br>1
MERS-CoV	0<br>4	0<br>3	0<br>3	0<br>3	8<br>0	0<br>3	1<br>0	0<br>1	0<br>4	0<br>3	10<br>11	1<br>2	1<br>1
SARS-CoV-Tor2	0<br>2	0<br>2	0<br>2	0<br>2	1<br>0	0<br>1	6<br>0	0<br>3	0<br>1	0<br>1	1<br>2	13<br>15	8<br>5

SARS-CoV-2	02	01	01	01	10	02	60	05	01	01	11	85	1112

*Functional families were calculated with Build Pangenome with OrthoMCL (KBaseGenomes).

Table S4. *Homologous families of Betacoronaviruses*

Genome	Homolog families*				
	MERS-CoV	SARS CoV-Tor2	SARS-CoV-2	MERS-CoV-Eng	SARS-CoV
MERS-CoV	8	1	1	8	1
SARS CoV-Tor2	1	8	8	1	6
SARS-CoV-2	1	8	8	1	6
MERS-CoV-Eng	8	1	1	9	2
SARS-CoV	1	6	6	2	7

*The functional families in betacoronaviruses were calculated with Build Pangenome with OrthoMCL (KBaseGenomes).

Table S5. *Identity analysis of the Nsp3*

**Type coronavirus**	**Accesion number**	**Identity (%) [a]**	**GMQE[b]**	**QMEAN[c]**
		Alphacoronavirus		
HCoVs-229E	NP 073549.1	42.32%	0.67	0.73 ± 0.05
HCoVs-NL63	YP 003766.2	42.32%	0.68	0.73 ± 0.05
		Betacoronavirus		
HCoVs-OC43	YP 009555238.1	29.24%	0.71	0.70 ± 0.05
HCoVs-HKU1	YP_173236.1	31.42%	0.65	0.69 ± 0.05
MERS-CoV	YP_009047215.1	100.00%	0.99	0.94 ± 0.05
MERS-CoV-Eng	YP_009944294.1	99.37%	0.98	0.94 ± 0.05
SARS-CoV	APO40578.1	80.52%	0.94	0.88 ± 0.05
SARS-CoV-2	YP_009725299.1	99.67%	0.98	0.91 ± 0.05
SARS-CoV-Tor2	NP_828862.2	82.41%	0.95	0.90 ± 0.05
		Gammacoronavirus		
AcCoV-Duck	YP_009825031.1	26.33%	0.53	0.58 ± 0.05
AcCoV- Turkey	YP_001941176.1	23.59%	0.54	0.58 ± 0.05
		Deltacoronavirus		
Porcine deltacoronavirus HKU15	QDH76194.1	20.20%	0.10	0.51 ± 0.08
Sparrow deltacoronavirus HKU17	AWV67106.1	96.03%	0.97	0.92 ± 0.07

aTarget–template sequence similarity is calculated from a normalised BLOSUM62 substitution matrix.
bQualitative model energy analysis.
cGlobal Model Quality Estimation.

Table S6. *Identity analysis of the Nsp5*

Type coronavirus	Accesion number	Identity (%) [a]	GMQE[b]	QMEAN[c]
		Alphacoronavirus		
HCoVs-229E	NP_073549.1	69.23%	0.89	0.85 ± 0.05
HCoVs-NL63	YP_003766.2	69.10%	0.90	0.87 ± 0.05
		Betacoronavirus		
HCoVs-OC43	YP_009924323.1	43.71%	0.81	0.80 ± 0.05
HCoVs-HKU1	YP_009944273.1	44.70%	0.82	0.82 ± 0.05
MERS-CoV	YP_009047217.1	47.68%	0.82	0.80 ± 0.05
MERS-CoV-Eng	YP_009944296.1	47.68%	0.82	0.80 ± 0.05
SARS-CoV	APO40578.1	95.30%	0.95	0.87 ± 0.05
SARS-CoV-2	YP_009725301.1	100.00%	0.95	0.88 ± 0.05
SARS-CoV-Tor2	NP_828849.7	100.00%	0.96	0.88 ± 0.05
		Gammacoronavirus		
AcCoV-Duck	YP_009825033.1	43.30%	0.78	0.77 ± 0.05
AcCoV- Turkey	YP_001941178.1	41.58%	0.78	0.79 ± 0.05
		Deltacoronavirus		
Porcine deltacoronavirus HKU15	QWE80491.1	37.88%	0.76	0.75 ± 0.05
Sparrow deltacoronavirus HKU17	AWV67106.1	38.23%	0.76	0.75 ± 0.05

aTarget–template sequence similarity is calculated from a normalised BLOSUM62 substitution matrix.

bQualitative model energy analysis.

cGlobal Model Quality Estimation.

Table S7. *Identity analysis of the Nsp6*

Type coronavirus	Accesion number	Identity (%) [a]	GMQE[b]	QMEAN[c]
		Alphacoronavirus		
HCoVs-229E	NP_073549.1	47.37%	0.00	0.47 ± 0.12
HCoVs-NL63	YP_003766.2	52.63%	0.00	0.39 ± 0.12
		Betacoronavirus		
HCoVs-OC43	YP_009555238.1	3.70%	0.01	0.34 ± 0.12
HCoVs-HKU1	YP_173236.1	7.41%	0.01	0.35 ± 0.12
MERS-CoV	YP_009047202.1	3.70%	0.01	0.30 ± 0.12
MERS-CoV-Eng	YP_007188577.3	3.70%	0.01	0.30 ± 0.12
SARS-CoV	APO40578.1	7.69%	0.00	0.33 ± 0.12
SARS-CoV-2	YP_009724389.1	7.69%	0.01	0.35 ± 0.12
SARS-CoV-Tor2	NP_828849.7	12.00%	0.00	0.17 ± 0.12
		Gammacoronavirus		
AcCoV-Duck	YP_009825007.1	7.41%	0.01	0.33 ± 0.12
AcCoV- Turkey	YP_001941164.2	11.11%	0.01	0.34 ± 0.12
		Deltacoronavirus		
Porcine deltacoronavirus HKU15	QWE80491.1	31.58%	0.00	0.28 ± 0.12
Sparrow deltacoronavirus HKU17	AWV67106.1	31.58%	0.00	0.33 ± 0.12

aTarget–template sequence similarity is calculated from a normalised BLOSUM62 substitution matrix.

bQualitative model energy analysis.

cGlobal Model Quality Estimation.

Table S8. *Identity analysis of the Nsp9*

Type coronavirus	Accesion number	Identity (%) [a]	GMQE[b]	QMEAN[c]
		Alphacoronavirus		
HCoVs-229E	NP_073549.1	45.87%	0.61	0.58 ± 0.08
HCoVs-NL63	YP_003766.2	49.54%	0.64	0.61 ± 0.08
		Betacoronavirus		
HCoVs-OC43	YP_009555238.1	46.36%	0.64	0.65 ± 0.06
HCoVs-HKU1	YP_173236.1	45.45%	0.67	0.64 ± 0.06
MERS-CoV	YP_009047202.1	52.73%	0.69	0.66 ± 0.06
MERS-CoV-Eng	YP_007188577.3	53.64%	0.70	0.65 ± 0.06
SARS-CoV	APO40578.1	98.23%	0.82	0.80 ± 0.06
SARS-CoV-2	YP_009724389.1	40.54%	0.68	0.68 ± 0.06
SARS-CoV-Tor2	NP_828849.7	100.00%	0.86	0.80 ± 0.06
		Gammacoronavirus		
AcCoV-Duck	YP_009825037.1	40.54%	0.68	0.68 ± 0.06
AcCoV- Turkey	YP_001941182.1	40.54%	0.66	0.66 ± 0.06
		Deltacoronavirus		
Porcine deltacoronavirus HKU15	QWE80491.1	36.11%	0.58	0.59 ± 0.06
Sparrow deltacoronavirus HKU17	AWV67106.1	35.19%	0.54	0.55 ± 0.06

aTarget–template sequence similarity is calculated from a normalised BLOSUM62 substitution matrix.

bQualitative model energy analysis.

cGlobal Model Quality Estimation.

Table S9. *Identity analysis of the RNA-dependent RNA polymerase Rdpd (Nsp12)*

**Type coronavirus**	**Accesion number**	**Identity (%)** [a]	**GMQE**[b]	**QMEAN**[c]
		Alphacoronavirus		
HCoVs-229E	NP_073549.1	58.86%	0.81	0.74 ± 0.05
HCoVs-NL63	YP_003766.2	58.96%	0.79	0.72 ± 0.05
		Betacoronavirus		
HCoVs-OC43	YP_009555260.1	72.73%	0.85	0.78 ± 0.05
HCoVs-HKU1	YP_459941.1	73.32%	0.84	0.77 ± 0.05
MERS-CoV	YP_009047223.1	75.71%	0.86	0.80 ± 0.05
MERS-CoV-Eng	YP_009944302.1	75.71%	0.86	0.80 ± 0.05
SARS-CoV	APO40578.1	95.68%	0.87	0.82 ± 0.05
SARS-CoV-2	YP_009725307.1	99.85%	0.88	0.83 ± 0.05
SARS-CoV-Tor2	NP_828849.7	96.57%	0.86	0.81 ± 0.05
		Gammacoronavirus		
AcCoV-Duck	YP_009825029.1	66.72%	0.81	0.73 ± 0.05
AcCoV- Turkey	YP_001941185.1	66.12%	0.80	0.71 ± 0.05
		Deltacoronavirus		
Porcine deltacoronavirus HKU15	QWE80491.1	52.41%	0.80	0.73 ± 0.05
Sparrow deltacoronavirus HKU17	AWV67106.1	53.28%	0.75	0.71 ± 0.05

aTarget–template sequence similarity is calculated from a normalised BLOSUM62 substitution matrix.

bQualitative model energy analysis.

cGlobal Model Quality Estimation.

Table S10. *Identity analysis of the Nsp13*

Type coronavirus	Accesion number	Identity (%) [a]	GMQE[b]	QMEAN[c]
		Alphacoronavirus		
HCoVs-229E	NP_073549.1	62.83%	0.78	0.76 ± 0.05
HCoVs-NL63	YP_003766.2	63.42%	0.79	0.76 ± 0.05
		Betacoronavirus		
HCoVs-OC43	YP_009555238.1	69.16%	0.77	0.75 ± 0.05
HCoVs-HKU1	YP_459942.1	68.26%	0.78	0.76 ± 0.05
MERS-CoV	YP_009047224.1	100.00%	0.74	0.67 ± 0.05
MERS-CoV-Eng	YP_009944303.1	99.83%	0.74	0.67 ± 0.05
SARS-CoV	APO40578.1	72.35%	0.82	0.81 ± 0.05
SARS-CoV-2	YP_009725308.1	73.01%	0.80	0.81 ± 0.05
SARS-CoV-Tor2	NP_828849.7	72.37%	0.81	0.81 ± 0.05
		Gammacoronavirus		
AcCoV-Duck	YP_009825025.1	62.43%	0.76	0.73 ± 0.05
AcCoV- Turkey	YP_001941186.1	59.88%	0.76	0.73 ± 0.05
		Deltacoronavirus		
Porcine coronavirus HKU15	QWE80491.1	58.10%	0.74	0.74 ± 0.05
Sparrow deltacoronavirus HKU17	AWV67106.1	58.41%	0.74	0.74 ± 0.05

aTarget–template sequence similarity is calculated from a normalised BLOSUM62 substitution matrix.

bQualitative model energy analysis.

cGlobal Model Quality Estimation.

Table S11. *Identity analysis of the Nsp14*

Type coronavirus	Accesion number	Identity (%) [a]	GMQE[b]	QMEAN[c]
		Alphacoronavirus		
HCoVs-229E	NP_073549.1	54.04%	0.80	0.72 ± 0.05
HCoVs-NL63	YP_003766.2	54.15%	0.79	0.72 ± 0.05
		Betacoronavirus		
HCoVs-OC43	YP_009555238.1	58.33%	0.81	0.73 ± 0.05
HCoVs-HKU1	YP_173236.1	57.56%	0.82	0.73 ± 0.05
MERS-CoV	YP_009047202.1	63.57%	0.82	0.75 ± 0.05
MERS-CoV-Eng	YP_007188577.3	63.57%	0.82	0.75 ± 0.05
SARS-CoV	APO40578.1	52.54%	0.78	0.71 ± 0.05
SARS-CoV-2	YP_009724389.1	93.67%	0.89	0.83 ± 0.05
SARS-CoV-Tor2	NP_828849.7	99.81%	0.90	0.84 ± 0.05
		Gammacoronavirus		
AcCoV-Duck	YP_009825006.1	52.34%	0.78	0.70 ± 0.05
AcCoV- Turkey	YP_001941164.2	99.81%		
		Deltacoronavirus		
Porcine coronavirus HKU15	QWE80491.1	43.82%	0.78	0.68 ± 0.05
Sparrow deltacoronavirus HKU17	AWV67106.1	43.82%	0.79	0.68 ± 0.05

aTarget–template sequence similarity is calculated from a normalised BLOSUM62 substitution matrix.

bQualitative model energy analysis.

cGlobal Model Quality Estimation.

Table S12. *Sequences included to obtain the consensus sequence of Nsp3.*

Dominio Conservado (CDD)	Conserved Protein Domain Family	Accesion number*	Length sequence consensus (aa)
cd21731	alphaCoV_PLPro (alphacoronavirus papain-like protease)	YP 003766.2, NP 073549.1, ALK43113.1, YP_009380519.1, APD51481.1, P0C6X5.1, ALK43114.1, P0C6X1.1, AYR18429.1, AYH64860.2, QER90703.1, AOV85633.1, QBP43277.1, QDF43809.1, QCX35166.1, P0C6V6.1, AIA62211.1, AIA62270.1, ABI14446.1, ASU62498.1, AMD11206.1, AZF86129.1, ASL24651.1, ADI80521.1, ASF90485.1, AIA62233.1, AZF86117.1, YP_009201729.1, AVY53334.1, AFU92112.1, ASL24650.1, AFU92077.1, AFH58022.1, BBA21166.1, YP_009380519.1	289
cd21732	betaCoV_PLPro (betacoronavirus papain-like protease)	YP 009555238.1, YP 173236.1, NP 828862.2, YP 009725299.1, YP 009047215.1, YP 009944294.1, YP 001941176.1, YP 001941176.1 YP_009555258.1, YP_009924321.1, YP_009742610.1, YP_009725299.1, YP_009742610.1, NP_828862.2, YP_009944368.1, YP_460024.1, YP_009944271.1, YP_009047215.1, YP_009047231.1, YP_009944294.1, YP_009944283.1, 3MJ5_A, QHD43415.1, 3MJ5_A, 5W8T_A, 4REZ_A, AYR18613.1, AVV62524.1, AVV62535.1, AYR18649.1, AUM60013.1, APO40578.1, AIG13097.1, AJA91204.1	304
cd21733	gammaCoV_PLPro (gammacoronavirus	YP_009825031.1, YP_001941176.1, AHB63480.1, YP_001876435.1, ABW75136.1, APP92719.1, QDA76254.1, AVI69518.1,	304

	papain-like protease)	ADV71748.1, CEK09107.1, QDY92334.1, QCB65096.1, QDQ69120.1, ACV87256.1, QDQ69094.1, AQY55829.1, AXB38893.1, ARJ35781.1, ADV71811.1, ALQ43513.1, AKF17723.1, ACV87267.1	
cd21734	deltaCoV_PLPro (deltacoronavirus papain-like protease)	QDH76194.1, YP_005352853.1, AXP32229.1, AWV96579.1, ATJ00129.1, BAZ95608.1, YP_005352845.1, AWV67124.1, AWV67133.1, BBC54860.1, BBC54851.1, AXP20280.1, YP_002308505.1, ACJ12043.1, YP_002308496.1, BBC54841.1, BBC54831.1, BBC54821.1, YP_005352862.1, AWR88311.1, AUG59154.1, YP_005352837.1, YP_005352880.1, YP_005352870.1	313

*Accession number of viral representatives of each taxonomic group was obtained from the NCBI (Conserved Protein Domain Family) database.

Table S13. *Sequences included to obtain the consensus sequence of Nsp5*

Dominio Conservado (CDD)	Conserved Protein Domain Family	Accesion number	Length sequence consensus (aa)
cd21665	alphaCoV_Nsp5_Mpro (alphacoronavirus non-structural protein 5, also called Main protease (Mpro)	NP_073549.1, YP_003766.2, 5GWY_A, 3TLO_A, 1LVO_A, 5NH0_A, 2ZU2_A, 1P9S_A, 5EU8_A, 4F49_A, 5ZQG_A, 4ZRO_A, ASF90469.1, QER90703.1, AID16674.1, AIA62211.1, AFU92103.1, AID16670.1, ASR18936.1, QCX35159.1, ADI80522.1, AID16673.1, APD51489.1, AZF86129.1, ABQ57215.1, AZF86117.1, AIA62270.1, AJP67437.1, ASL24651.1, ATP66788.1, ATP66783.1, QBP43277.1	296
d21666	betaCoV_Nsp5_Mpro (betacoronavirus non-structural protein 5, also called Main protease (Mpro)	APO40578.1, YP_009725301.1, NP_828849.7, YP_009924323.1, YP_009944273.1, YP_009047217.1, YP_009944296.1, 3D23_B, 6M2N_A, 6JIJ_B, 2A5K_A, 1WOF_A, 3ATW_A, 4MDS_A, 4YLU_A, 5WKJ_A, 1Q2W_A, 1UJ1_A, 1Z1J_A, 2PWX_A, 2Q6G_A, 3E91_A, 3M3T_A, 4HI3_A, 5B6O_A, AID16629.1, AGT28265.1, QGA70691.1, AOG30811.1,	297

		ADY69163.1, YP_009072438.1, AWV67038.1, 6JIJ_B, 2YNA	
cd21667	gammaCoV_Nsp5_Mpro (gammacoronavirus non-structural protein 5, also called Main protease (Mpro)	YP_009825033.1, YP_001941178.1, 2Q6D_A, QCE31532.1, AAW33784.1, AZP23929.1, AHB63507, QCB65096.1, AKQ98469.1, AKQ98473.1, NP_040829.1, NP_066134.1, QEG79146.1, ALQ43513.1, CAC39112.1, ACV87243.1, ANY58999.1, ARJ35781.1, QDA76254.1, QDA76263.1, ABW75124.1, ARJ35790.1, CAZ86698.1, CAZ86697.1, CEK09106.1, NP_740623.1, AKQ98471.1, AKF17722.1, ATE90965.1, ATE90966.1, QCB65092.1, YP_001941178.1, AHB63480.1, YP_001876436.1, AXF38647.1	306
cd21668	deltaCoV_Nsp5_Mpro (deltacoronavirus non-structural protein 5, also called Main protease (Mpro)	BBC54821.1, YP_005352862.1, AWR88311.1, APZ76696.1, ACJ12043.1, YP_005352880.1, YP_005352870.1	302

*Accession number of viral representatives of each taxonomic group was obtained from the NCBI (Conserved Protein Domain Family) database.

Table S14. *Sequences included to obtain the consensus sequence of Nsp6*

**Dominio Conservado (CDD)**	**Conserved Protein Domain Family**	**Accesion number**	**Length sequence consensus (aa)**
cd21558	alphaCoV-Nsp6 (alphacoronavirus non-structural protein 6)	NP_073549.1, YP_003766.2, ANA96047.1, AID16670.1, AIA62270.1, QBP43254.1, AFU92103.1, ALK43113.1, YP_009328933.1, APD51481.1, AYR18486.1, AID16674.1, QER90703.1, QCX35177.1, QBP43266.1, QBP43288.1, YP_009199608.1, ACA52156.1, ASL24651.1, ARI44793.1, QCX35159.1, AIA62233.1, AID16673.1, AVM80482.1, AIA62211.1, YP_009201729.1, ADI80522.1, AZF86117.1, AZF86129.1, ATP66737.1, YP_009380519.1, ACT10853.1, ATP66783.1, AVM87573.1, ASF90443.1, ASF90485.1, ASF90469.1	293
cd21560	betaCoV-Nsp6 (betacoronavirus non-structural protein 6)	YP_009555238.1, YP_173236.1, YP_009047202.1, YP_007188577.3, YP_009724389.1, NP_828849.7, APO40578.1, YP_009742613.1, ATP66760.1, ASL68951.1, YP_009361855.1, APO40578.1, ATQ39389.1, AUM60023.1, AVV62524.1, YP_003858583.1, ATP66754.1, AYR18613.1,	290

		BBJ36006.1, ATP66777.1, ABN10918.1, ADM33573.1, QEH60462.1, QGA70691.1, AOG30811.1, AWV67038.1, ADY69163.1, AJA91204.1, AFE48810.1, ATP66742.1, AGT28265.1, AVP25405.1, YP_009072438.1	
cd21559	gammaCoV-Nsp6	YP_009825007.1, YP_001941164.2, QDY92358.1, QDY92334.1, QEG79146.1, QCB65096.1, AHB63480.1, QDQ69197.1, QDQ69171.1	307
cd21561	deltaCoV-Nsp6	YP_009513020.1, YP_005352837.1, YP_005352880.1, AMN91620.1, YP_002308505.1, BBC54821.1, AWV67106.1, YP_002308496.1, ACJ12043.1, YP_002308478.1, AWV67124.1, YP_005352845.1, YP_005352853.1, AXP20280.1, AWR88311.1, YP_005352870.1	296

*Accession number of viral representatives of each taxonomic group was obtained from the NCBI (Conserved Protein Domain Family) database.

***Table S15**. Sequences included to obtain the consensus sequence of Nsp9*

Dominio Conservado (CDD)	Conserved Protein Domain Family	Accesion number	Length sequence consensus (aa)
cd21897	CoV-Nsp9 (coronavirus non-structural protein 9)	YP_003766.2, NP_073549.1, AID16671.1, ASF90443.1, ASL24651.1, AZF86129.1, YP_009201729.1, ANA96047.1, QBP43288.1, AZF86117.1, QBP43266.1, AID16669.1, QCX35166.1, AOG74788.1, AFU92077.1, AID16674.1, APD51505.1, APD51497.1, ALK43115.1, ALK43113.1, AJP67413.1, ACI13472.1, QER90703.1, ACI13440.1, APD51489.1, AYH64861.2, QCX35159.1, AID16673.1, ARI44793.1, AYR18560.1, AYR18498.1, QDM36985.1, YP_009200734.1, AVM80473.1, AID16670.1, ASF90459.1, AGZ84525.1, ASF90469.1, ACT10915.1, YP_009256195.1, BBA21166.1, ATP66737.1, YP_009380519.1, QDF43809.1, AIA62211.1, AIA62226.1, AIA62205.1, ACA52156.1, AVY53334.1	108
cd21898	betaCoV_Nsp9 (betacoronavirus non-structural protein 9)	YP_009555238.1, YP_173236.1, YP_009047202.1, YP_007188577.3,	111

		YP_009724389.1, NP_828849.7, APO40578.1, YP_009072438.1, BBJ36006.1, ASL68940.1, ANA96038.1, AVV62524.1, AVV62535.1, ABN10918.1, QGA70691.1, QEH60462.1, AVP25405.1, AGT28265.1, AOG30811.1, AWV67038.1, AID16712.1, ATP66754.1, ATP66760.1, ATP66742.1, ATP66748.1, ATP66777.1, ATP66731.1, AZU96324.1	
cd21899	gammaCoV_Nsp9 (gammacoronavirus non-structural protein 9)	YP_009825037.1, YP_001941182.1, AHB63480.1, AFJ11174.1, QCB65092.1, AGK85497.1, ARS22408.1, AEP84744.1, QDY92334.1, ADV71798.1, QDA76290.1	113
cd21900	deltaCoV_Nsp9 (deltacoronavirus non-structural protein 9)	YP_005352862.1, AWR88311.1, AXP20280.1, AWV67124, YP_002308505, ACJ12043	109

*Accession number of viral representatives of each taxonomic group was obtained from the NCBI (Conserved Protein Domain Family) database.

Table S16. *Sequences included to obtain the consensus sequence of RNA-dependent RNA polymerase Rdpd (Nsp12)*

Dominio Conservado (CDD)	Conserved Protein Domain Family	Accesion number	Length sequence consensus (aa)
cd21588	alphaCoV_RdRp	YP 003766.2, NP 073549.1, AVA26872.1, AIA62219.1, AXE71622.1, ADX59465.1, ADX59457.1, QBP43277.1, QCX35166.1, AZF86117.1, QDF43809.1, YP_009201729.1, AIA62270.1, YP_009199608.1, AIA62199.1, ASF90474.1, ASF90495.1, ASF90459.1, ASF90443.1, ARI44788.1, AID16673.1, APD51489.1, AFU92130.1, AMC39583.1, QBP43288.1, AKJ21970.1, AZF86129.1, Q98VG9.2 (R1AB_FIPV), ANA96047.1, P0C6Y5.1 (R1AB_CVPPU), AJO27002.1, AVY53334.1, AYR18560.1, QCX35159.1, ABQ57215.1, ALK43113.1, ADX59494.1, APD51505.1, AIA62211.1, AYF53092.1, YP_009328933.1, YP_009256195.1, ADX59487.1, AID16670.1, AMB42807.1, AID16669.1, AKZ66481.1, ACA52156.1, QDF43789.1, AYH64861.2, NP_073549.1, AID16547.1, ASL24651.1, QER90703.1, ADX59501.1, ABB77038.1, QBM00060.1, AQK38167.1, ATP66737.1, ATP66788.1, ATP66783.1	924
cd21589	betaCoV_RdRp	NP 073549.1, YP 009944302.1, YP 009725307.1, NP 828849.7, APO40578.1, YP 009555260.1, YP 459941.1, 7BTF_A,	929

		P0C6X4.1 (R1AB_CVHN5), YP_009072438.1, ATQ39389.1, ABN10934.1	
cd21587	gammaCoV_RdRp	YP 459941.1, YP 009825029.1, AHB73997.1, P0C6Y3.1 (R1AB_IBVM), AVI69484.1, ACH72802.1, P0C6Y1.1 (R1AB_IBVB), ACC94324.1, ABW75136.1, AKQ98476.1, AAP92673.1, ACV87266.1 QDA76263.1, ARB66179.1, AXB38892.1, APZ73705.1, ARS23151.1, AIG54294.1, AOC59721.1, QDY92334.1, AAQ21583.1, AMD40261.1, AXT92414.1, QDQ69132.1, QDQ69106.1, APP92719.1, QDQ69145.1, AKQ98478.1, QDQ69158.1, AGW81826.1, AKF17722.1, ARJ35781.1, AJT47940.1, AGK85497.1, APY23514.1, APP92727.1, QCB65096.1, CAA83018.1, AKV63203.1, AVI69518.1, AHB63507.1	931
cd21590	deltaCoV_RdRp	AWV67106.1, AWV67124.1, BBC54860.1, ANI85845.1, ACJ12043.1, BBC54841.1, YP_002308496.1, AWV67133.1, YP_002308505.1, AXP20280.1 AML40817.1, BBC54831.1, ASR75138.1, YP_005352845.1, AMN91620.1, BBC54851.1, YP_005352862.1, YP_005352853.1, YP_005352837.1, YP_005352880.1, YP_002308478.1, YP_005352870.1, AWV96565.1	928

*Accession number of viral representatives of each taxonomic group was obtained from the NCBI (Conserved Protein Domain Family) database.

Table S17. *Sequences included to obtain the consensus sequence of Nsp13*

**Dominio Conservado (CDD)**	**Conserved Protein Domain Family**	**Accesion number**	**Length sequence consensus (aa)**
cd21723	alphaCoV_Nsp13-helicase (helicase domain of alphacoronavirus non-structural protein 13)	NP_073549.1, YP_003766.2 P0C6X5.1, QBP43288.1, APD51489.1, YP_009328933.1, QDF43809.1, AIA62199.1, AFU92112.1, ADX59465.1, ADX59494.1, AYR18560.1, AEK25510.1, ADX59501.1, QER90703.1, AYH64861.2, ADX59457.1, ARI44788.1, ADX59487.1, AVY53334.1, AIA62270.1, BAV31348.1, ATP66737.1, ABB77061.1, AVM80473.1, ASL24651.1, AIA62233.1, AZF86117.1, AKZ66481.1, YP_009201729.1, ABO88147.1, YP_009380520.1, AYR18518.1, AID16669.1, ASF90474.1, ATP66783.1	340
cd21722	betaCoV_Nsp13-helicase (helicase domain of betacoronavirus non-structural protein 13)	YP_009555238.1, YP_459942.1, YP_009047224.1, YP_009944303.1, YP_009725308.1, APO40578.1, NP_828849.7, QHD43415.1, AUM60023.1, AYR18640.1, AYR18613.1, 6JYT_A, 5WWP_B, ADY69164.1, QDF43839.1, YP_009072438.1, AOG30811.1, BBJ36006.1,	340

		ABN10892.1, AUM60023.1, ADX59473.1, QGA70691.1, QCC20711.1, ATP66731.1, AYR18596.1, ATP66742.1, AFE48822.1	
cd21720	gammaCoV_Nsp13-helicase (helicase domain of gammacoronavirus non-structural protein 13)	YP_009825025.1, YP_001941186.1, AHB63507.1, AKF17722.1, QCB65096.1, AGK85497.1, AKQ98482.1, APY26727.1, ADP06517.1, ADV71748.1, AKV63203.1	343
cd21721	deltaCoV_Nsp13-helicase (helicase domain of deltacoronavirus non-structural protein 13)	ACJ12043.1, YP_002308496.1, YP_005352837.1, YP_002308505.1, YP_005352880.1, BBC54821.1, AWV67106.1, YP_005352870.1, YP_005352862.1, AWR88314.1	342

*Accession number of viral representatives of each taxonomic group was obtained from the NCBI (Conserved Protein Domain Family) database.

Table S18. *Sequences included to obtain the consensus sequence of Nsp14*

Dominio Conservado (CDD)	Conserved Protein Domain Family	Accesion number	Length sequence consensus (aa)
cd21660	alphaCoV_Nsp14 (nonstructural protein 14 of alphacoronavirus)	NP_073549.1, YP_003766.2 YP_009380520.1, ACA52156.1, AID16673.1, AFU92103.1, YP_009328933.1, ANA96047.1, ASL24651.1, AIA62233.1, AYR18435.1, ADR79379.1, AYH64858.2, AID16669.1, APD51497.1, ADX59457.1, AZF86123.1, AZF86117.1, APD51481.1, AYR18399.1, QEH62668.1, ADX59465.1, AID16674.1, BBA21167.1, ADX59501.1, QED88031.1, QBP43266.1, QCX35159.1, AVY53334.1, ATP66783.1. ATP66788.1, QER90703.1, AIA62270.1, AZF86129.1, AHM88216.1, YP_009201729.1, ALK43113.1, AIA62199.1, NP_073549.1, QBP43288.1, YP_009199608.1, ASF90459.1, ADX59494.1, YP_009019180.1	510
cd21659	betaCoV_Nsp14 (nonstructural protein 14 of betacoronavirus)	YP_009555238.1, YP_173236.1, YP_009047202.1, YP_007188577.3, YP_009724389.1, APO40578.1, NP_828849.7, ADY69164.1, ASU45783.1, YP_009072438.1,	519

		AGT28265.1, AOG30811.1, ABN10934.1, AWV67063.1, ATP66748.1, ATP66731.1, BAS18842.1, BAS18842.1, AUM60013.1, AIA62342.1, BBJ36006.1, ASL68951.1, AVV62535.1, ADM33557.1, QGA70691.1, AVP25405.1, YP_003858583.1, ADX59481.1, APO40578.1, ATP66725.1, ATP66760.1, AYR18613.1, AYR18596.1, AID16659.1, ATP66742.1, AAA46458.2, AIA62351.1, AZU96325.1, QDF43839.1	
cd21658	gammaCoV_Nsp14 (nonstructural protein 14 of gammacoronavirus)	YP_009825006.1, YP_001941164.2, QCB65096.1, AHB63507.1, AIG54293.1, APP92727.1, QDQ69197.1, CEK09107.1, ABW75124.1, QDA76254.1, AXQ05189.1, ALQ43514.1, ABW75136.1, QDY92334.1, AKQ98488.1	518
cd21657	deltaCoV_Nsp14 (nonstructural protein 14 of deltacoronavirus)	ACJ12043.1, YP_005352862.1, BBC54821.1, YP_005352870.1, YP_002308505.1, AXP20280.1, YP_005352853.1, YP_005352837.1, AWV67124.1, BBC54851.1, YP_002308496.1, AVR48517.1, AWV96579.1, ABQ39957.1, AWV96586.1	508

Table S19. *Sequences included to obtain consensus sequence the non-structural protein of coronavirus*

Non structural protein	Conserved Protein Domain Family	Dominio Conservado (CDD)	Accesion number	Length sequence consensus (aa)
		Alphacoronavirus		
Nsp3	alphaCoV_PLPro (alphacoronavirus papain-like protease)	cd21731	YP 003766.2, NP 073549.1, ALK43113.1, YP_009380519.1, APD51481.1, P0C6X5.1, ALK43114.1, P0C6X1.1, AYR18429.1, AYH64860.2, QER90703.1, AOV85633.1, QBP43277.1, QDF43809.1, QCX35166.1, P0C6V6.1, AIA62211.1, AIA62270.1, ABI14446.1, ASU62498.1, AMD11206.1, AZF86129.1, ASL24651.1, ADI80521.1, ASF90485.1, AIA62233.1, AZF86117.1, YP_009201729.1, AVY53334.1, AFU92112.1, ASL24650.1, AFU92077.1, AFH58022.1, BBA21166.1, YP_009380519.1	289
Nsp5	alphaCoV_Nsp5_Mpro (alphacoronavirus non-structural protein 5, also called Main protease (Mpro)	cd21665	NP_073549.1, YP_003766.2, 5GWY_A, 3TLO_A, 1LVO_A, 5NH0_A, 2ZU2_A, 1P9S_A, 5EU8_A, 4F49_A, 5ZQG_A, 4ZRO_A, ASF90469.1, QER90703.1, AID16674.1, AIA62211.1, AFU92103.1, AID16670.1, ASR18936.1, QCX35159.1, ADI80522.1, AID16673.1, APD51489.1, AZF86129.1, ABQ57215.1, AZF86117.1, AIA62270.1, AJP67437.1, ASL24651.1, ATP66788.1, ATP66783.1, QBP43277.1	296
Nsp6	alphaCoV-Nsp6 (alphacoronavirus non-structural protein 6)	cd21558	NP_073549.1, YP_003766.2, ANA96047.1, AID16670.1, AIA62270.1, QBP43254.1, AFU92103.1, ALK43113.1, YP_009328933.1, APD51481.1, AYR18486.1, AID16674.1, QER90703.1, QCX35177.1, QBP43266.1, QBP43288.1, YP_009199608.1, ACA52156.1, ASL24651.1, ARI44793.1, QCX35159.1, AIA62233.1, AID16673.1, AVM80482.1, AIA62211.1, YP_009201729.1, ADI80522.1, AZF86117.1, AZF86129.1, ATP66737.1, YP_009380519.1, ACT10853.1, ATP66783.1, AVM87573.1, ASF90443.1, ASF90485.1, ASF90469.1	293
Nsp9	CoV-Nsp9 (coronavirus non-structural protein 9)	cd21897	YP_003766.2, NP_073549.1, AID16671.1, ASF90443.1, ASL24651.1, AZF86129.1, YP_009201729.1, ANA96047.1, QBP43288.1, AZF86117.1, QBP43266.1, AID16669.1, QCX35166.1, AOG74788.1, AFU92077.1, AID16674.1, APD51505.1, APD51497.1, ALK43115.1, ALK43113.1, AJP67413.1, ACI13472.1, QER90703.1, ACI13440.1, APD51489.1, AYH64861.2, QCX35159.1, AID16673.1, ARI44793.1, AYR18560.1, AYR18498.1, QDM36985.1, YP_009200734.1, AVM80473.1, AID16670.1, ASF90459.1, AGZ84525.1, ASF90469.1, ACT10915.1, YP_009256195.1, BBA21166.1, ATP66737.1,	108

			YP_009380519.1, QDF43809.1, AIA62211.1, AIA62226.1, AIA62205.1, ACA52156.1, AVY53334.1	
Nsp12*	alphaCoV_RdRp	cd21588	YP 003766.2, NP 073549.1, AVA26872.1, AIA62219.1, AXE71622.1, ADX59465.1, ADX59457.1, QBP43277.1, QCX35166.1, AZF86117.1, QDF43809.1, YP_009201729.1, AIA62270.1, YP_009199608.1, AIA62199.1, ASF90474.1, ASF90495.1, ASF90459.1, ASF90443.1, ARI44788.1, AID16673.1, APD51489.1, AFU92130.1, AMC39583.1, QBP43288.1, AKJ21970.1, AZF86129.1, Q98VG9.2 (R1AB_FIPV), ANA96047.1, P0C6Y5.1 (R1AB_CVPPU), AJO27002.1, AVY53334.1, AYR18560.1, QCX35159.1, ABQ57215.1, ALK43113.1, ADX59494.1, APD51505.1, AIA62211.1, AYF53092.1, YP_009328933.1, YP_009256195.1, ADX59487.1, AID16670.1, AMB42807.1, AID16669.1, AKZ66481.1, ACA52156.1, QDF43789.1, AYH64861.2, NP_073549.1, AID16547.1, ASL24651.1, QER90703.1, ADX59501.1, ABB77038.1, QBM00060.1, AQK38167.1, ATP66737.1, ATP66788.1, ATP66783.1	924
Nsp13	alphaCoV_Nsp13-helicase (helicase domain of alphacoronavirus non-structural protein 13)	cd21723	NP_073549.1, YP_003766.2, P0C6X5.1, QBP43288.1, APD51489.1, YP_009328933.1, QDF43809.1, AIA62199.1, AFU92112.1, ADX59465.1, ADX59494.1, AYR18560.1, AEK25510.1, ADX59501.1, QER90703.1, AYH64861.2, ADX59457.1, ARI44788.1, ADX59487.1, AVY53334.1, AIA62270.1, BAV31348.1, ATP66737.1, ABB77061.1, AVM80473.1, ASL24651.1, AIA62233.1, AZF86117.1, AKZ66481.1, YP_009201729.1, ABO88147.1, YP_009380520.1, AYR18518.1, AID16669.1, ASF90474.1, ATP66783.1	340
Nsp14	alphaCoV_Nsp14 (nonstructural protein 14 of alphacoronavirus)	cd21660	NP_073549.1, YP_003766.2, YP_009380520.1, ACA52156.1, AID16673.1, AFU92103.1, YP_009328933.1, ANA96047.1, ASL24651.1, AIA62233.1, AYR18435.1, ADR79379.1, AYH64858.2, AID16669.1, APD51497.1, ADX59457.1, AZF86123.1, AZF86117.1, APD51481.1, AYR18399.1, QEH62668.1, ADX59465.1, AID16674.1, BBA21167.1, ADX59501.1, QED88031.1, QBP43266.1, QCX35159.1, AVY53334.1, ATP66783.1. ATP66788.1, QER90703.1, AIA62270.1, AZF86129.1, AHM88216.1, YP_009201729.1, ALK43113.1, AIA62199.1, NP_073549.1, QBP43288.1, YP_009199608.1, ASF90459.1, ADX59494.1, YP_009019180.1	510
		Betacoronavirus		
Nsp3	betaCoV_PLPro	cd21732	YP 009555238.1, YP 173236.1, NP 828862.2, YP 009725299.1, YP 009047215.1, YP 009944294.1, YP 001941176.1, YP 001941176.1	304

	(betacoronavirus papain-like protease)		YP_009555258.1, YP_009924321.1, YP_009742610.1, YP_009725299.1, YP_009742610.1, NP_828862.2, YP_009944368.1, YP_460024.1, YP_009944271.1, YP_009047215.1, YP_009047231.1, YP_009944294.1, YP_009944283.1, 3MJ5_A, QHD43415.1, 3MJ5_A, 5W8T_A, 4REZ_A, AYR18613.1, AVV62524.1, AVV62535.1, AYR18649.1, AUM60013.1, APO40578.1, AIG13097.1, AJA91204.1	
Nsp5	betaCoV_Nsp5_Mpro (betacoronavirus non-structural protein 5, also called Main protease (Mpro)	d21666	APO40578.1, YP_009725301.1, NP_828849.7, YP_009924323.1, YP_009944273.1, YP_009047217.1, YP_009944296.1, 3D23_B, 6M2N_A, 6JIJ_B, 2A5K_A, 1WOF_A, 3ATW_A, 4MDS_A, 4YLU_A, 5WKJ_A, 1Q2W_A, 1UJ1_A, 1Z1J_A, 2PWX_A, 2Q6G_A, 3E91_A, 3M3T_A, 4HI3_A, 5B6O_A, AID16629.1, AGT28265.1, QGA70691.1, AOG30811.1, ADY69163.1, YP_009072438.1, AWV67038.1, 6JIJ_B, 2YNA	297
Nsp6	betaCoV-Nsp6 (betacoronavirus non-structural protein 6)	cd21560	YP_009555238.1, YP_173236.1, YP_009047202.1, YP_007188577.3, YP_009724389.1, NP_828849.7, APO40578.1, YP_009742613.1, ATP66760.1, ASL68951.1, YP_009361855.1, APO40578.1, ATQ39389.1, AUM60023.1, AVV62524.1, YP_003858583.1, ATP66754.1, AYR18613.1, BBJ36006.1, ATP66777.1, ABN10918.1, ADM33573.1, QEH60462.1, QGA70691.1, AOG30811.1, AWV67038.1, ADY69163.1, AJA91204.1, AFE48810.1, ATP66742.1, AGT28265.1, AVP25405.1, YP_009072438.1	290
Nsp9	betaCoV_Nsp9 (betacoronavirus non-structural protein 9)	cd21898	YP_009555238.1, YP_173236.1, YP_009047202.1, YP_007188577.3, YP_009724389.1, NP_828849.7, APO40578.1, YP_009072438.1, BBJ36006.1, ASL68940.1, ANA96038.1, AVV62524.1, AVV62535.1, ABN10918.1, QGA70691.1, QEH60462.1, AVP25405.1, AGT28265.1, AOG30811.1, AWV67038.1, AID16712.1, ATP66754.1, ATP66760.1, ATP66742.1, ATP66748.1, ATP66777.1, ATP66731.1, AZU96324.1	111
Nsp12*	betaCoV_RdRp	cd21589	NP 073549.1, YP 009944302.1, YP 009725307.1, NP 828849.7, APO40578.1, YP 009555260.1, YP 459941.1, 7BTF_A, P0C6X4.1 (R1AB_CVHN5), YP_009072438.1, ATQ39389.1, ABN10934.1	929
Nsp13	betaCoV_Nsp13-helicase (helicase domain of betacoronavirus non-structural protein 13)	cd21722	YP_009555238.1, YP_459942.1, YP_009047224.1, YP_009944303.1, YP_009725308.1, APO40578.1, NP_828849.7, QHD43415.1, AUM60023.1, AYR18640.1, AYR18613.1, 6JYT_A, 5WWP_B, ADY69164.1, QDF43839.1, YP_009072438.1, AOG30811.1, BBJ36006.1, ABN10892.1, AUM60023.1, ADX59473.1, QGA70691.1, QCC20711.1, ATP66731.1, AYR18596.1, ATP66742.1, AFE48822.1	340

Nsp14	betaCoV_Nsp14 (nonstructural protein 14 of betacoronavirus)	cd21659	YP_009555238.1, YP_173236.1, YP_009047202.1, YP_007188577.3, YP_009724389.1, APO40578.1, NP_828849.7, ADY69164.1, ASU45783.1, YP_009072438.1, AGT28265.1, AOG30811.1, ABN10934.1, AWV67063.1, ATP66748.1, ATP66731.1, BAS18842.1, BAS18842.1, AUM60013.1, AIA62342.1, BBJ36006.1, ASL68951.1, AVV62535.1, ADM33557.1, QGA70691.1, AVP25405.1, YP_003858583.1, ADX59481.1, APO40578.1, ATP66725.1, ATP66760.1, AYR18613.1, AYR18596.1, AID16659.1, ATP66742.1, AAA46458.2, AIA62351.1, AZU96325.1, QDF43839.1	519
		Gammacoronavirus		
Nsp3	gammaCoV_PLPro (gammacoronavirus papain-like protease)	cd21733	YP_009825031.1, YP_001941176.1, AHB63480.1, YP_001876435.1, ABW75136.1, APP92719.1, QDA76254.1, AVI69518.1, ADV71748.1, CEK09107.1, QDY92334.1, QCB65096.1, QDQ69120.1, ACV87256.1, QDQ69094.1, AQY55829.1, AXB38893.1, ARJ35781.1, ADV71811.1, ALQ43513.1, AKF17723.1, ACV87267.1	304
Nsp5	gammaCoV_Nsp5_Mpro (gammacoronavirus non-structural protein 5, also called Main protease (Mpro)	cd21667	YP_009825033.1, YP_001941178.1, 2Q6D_A, QCE31532.1, AAW33784.1, AZP23929.1, AHB63507, QCB65096.1, AKQ98469.1, AKQ98473.1, NP_040829.1, NP_066134.1, QEG79146.1, ALQ43513.1, CAC39112.1, ACV87243.1, ANY58999.1, ARJ35781.1, QDA76254.1, QDA76263.1, ABW75124.1, ARJ35790.1, CAZ86698.1, CAZ86697.1, CEK09106.1, NP_740623.1, AKQ98471.1, AKF17722.1, ATE90965.1, ATE90966.1, QCB65092.1, YP_001941178.1, AHB63480.1, YP_001876436.1, AXF38647.1	306
Nsp6	gammaCoV-Nsp6	cd21559	YP_009825007.1, YP_001941164.2, QDY92358.1, QDY92334.1, QEG79146.1, QCB65096.1, AHB63480.1, QDQ69197.1, QDQ69171.1	307
Nsp9	gammaCoV_Nsp9 (gammacoronavirus non-structural protein 9)	cd21899	YP_009825037.1, YP_001941182.1, AHB63480.1, AFJ11174.1, QCB65092.1, AGK85497.1, ARS22408.1, AEP84744.1, QDY92334.1, ADV71798.1, QDA76290.1	113
Nsp12*	gammaCoV_RdRp	cd21587	YP 459941.1, YP 009825029.1, AHB73997.1, P0C6Y3.1 (R1AB_IBVM), AVI69484.1, ACH72802.1, P0C6Y1.1 (R1AB_IBVB), ACC94324.1, ABW75136.1, AKQ98476.1, AAP92673.1, ACV87266.1 QDA76263.1, ARB66179.1, AXB38892.1, APZ73705.1, ARS23151.1, AIG54294.1, AOC59721.1, QDY92334.1, AAQ21583.1, AMD40261.1, AXT92414.1, QDQ69132.1, QDQ69106.1, APP92719.1, QDQ69145.1, AKQ98478.1, QDQ69158.1, AGW81826.1, AKF17722.1, ARJ35781.1, AJT47940.1, AGK85497.1, APY23514.1, APP92727.1, QCB65096.1, CAA83018.1, AKV63203.1, AVI69518.1, AHB63507.1	931

Nsp13	gammaCoV_Nsp13-helicase (helicase domain of gammacoronavirus non-structural protein 13)	cd21720	YP_009825025.1, YP_001941186.1, AHB63507.1, AKF17722.1, QCB65096.1, AGK85497.1, AKQ98482.1, APY26727.1, ADP06517.1, ADV71748.1, AKV63203.1	343
Nsp14	gammaCoV_Nsp14 (nonstructural protein 14 of gammacoronavirus)	cd21658	YP_009825006.1, YP_001941164.2, QCB65096.1, AHB63507.1, AIG54293.1, APP92727.1, QDQ69197.1, CEK09107.1, ABW75124.1, QDA76254.1, AXQ05189.1, ALQ43514.1, ABW75136.1, QDY92334.1, AKQ98488.1	518
			Deltacoronavirus	
Nsp3	deltaCoV_PLPro (deltacoronavirus papain-like protease)	cd21734	QDH76194.1, YP_005352853.1, AXP32229.1, AWV96579.1, ATJ00129.1, BAZ95608.1, YP_005352845.1, AWV67124.1, AWV67133.1, BBC54860.1, BBC54851.1, AXP20280.1, YP_002308505.1, ACJ12043.1, YP_002308496.1, BBC54841.1, BBC54831.1, BBC54821.1, YP_005352862.1, AWR88311.1, AUG59154.1, YP_005352837.1, YP_005352880.1, YP_005352870.1	313
Nsp5	deltaCoV_Nsp5_Mpro (deltacoronavirus non-structural protein 5, also called Main protease (Mpro)	cd21668	BBC54821.1, YP_005352862.1, AWR88311.1, APZ76696.1, ACJ12043.1, YP_005352880.1, YP_005352870.1	302
Nsp6	deltaCoV-Nsp6	cd21561	YP_009513020.1, YP_005352837.1, YP_005352880.1, AMN91620.1, YP_002308505.1, BBC54821.1, AWV67106.1, YP_002308496.1, ACJ12043.1, YP_002308478.1, AWV67124.1, YP_005352845.1, YP_005352853.1, AXP20280.1, AWR88311.1, YP_005352870.1	296
Nsp9	deltaCoV_Nsp9 (deltacoronavirus non-structural protein 9)	cd21900	YP_005352862.1, AWR88311.1, AXP20280.1, AWV67124, YP_002308505, ACJ12043	109
Nsp12*	deltaCoV_RdRp	cd21590	AWV67106.1, AWV67124.1, BBC54860.1, ANI85845.1, ACJ12043.1, BBC54841.1, YP_002308496.1, AWV67133.1, YP_002308505.1, AXP20280.1, AML40817.1, BBC54831.1, ASR75138.1, YP_005352845.1, AMN91620.1, BBC54851.1, YP_005352862.1, YP_005352853.1, YP_005352837.1, YP_005352880.1, YP_002308478.1, YP_005352870.1, AWV96565.1	928

Nsp13	deltaCoV_Nsp13-helicase (helicase domain of deltacoronavirus non-structural protein 13)	cd21721	ACJ12043.1, YP_002308496.1, YP_005352837.1, YP_002308505.1, YP_005352880.1, BBC54821.1, AWV67106.1, YP_005352870.1, YP_005352862.1, AWR88314.1	342
Nsp14	deltaCoV_Nsp14 (nonstructural protein 14 of deltacoronavirus)	cd21657	ACJ12043.1, YP_005352862.1, BBC54821.1, YP_005352870.1, YP_002308505.1, AXP20280.1, YP_005352853.1, YP_005352837.1, AWV67124.1, BBC54851.1, YP_002308496.1, AVR48517.1, AWV96579.1, ABQ39957.1, AWV96586.1	508

*Accession number of viral representatives of each taxonomic group was obtained from the NCBI (Conserved Protein Domain Family) database.

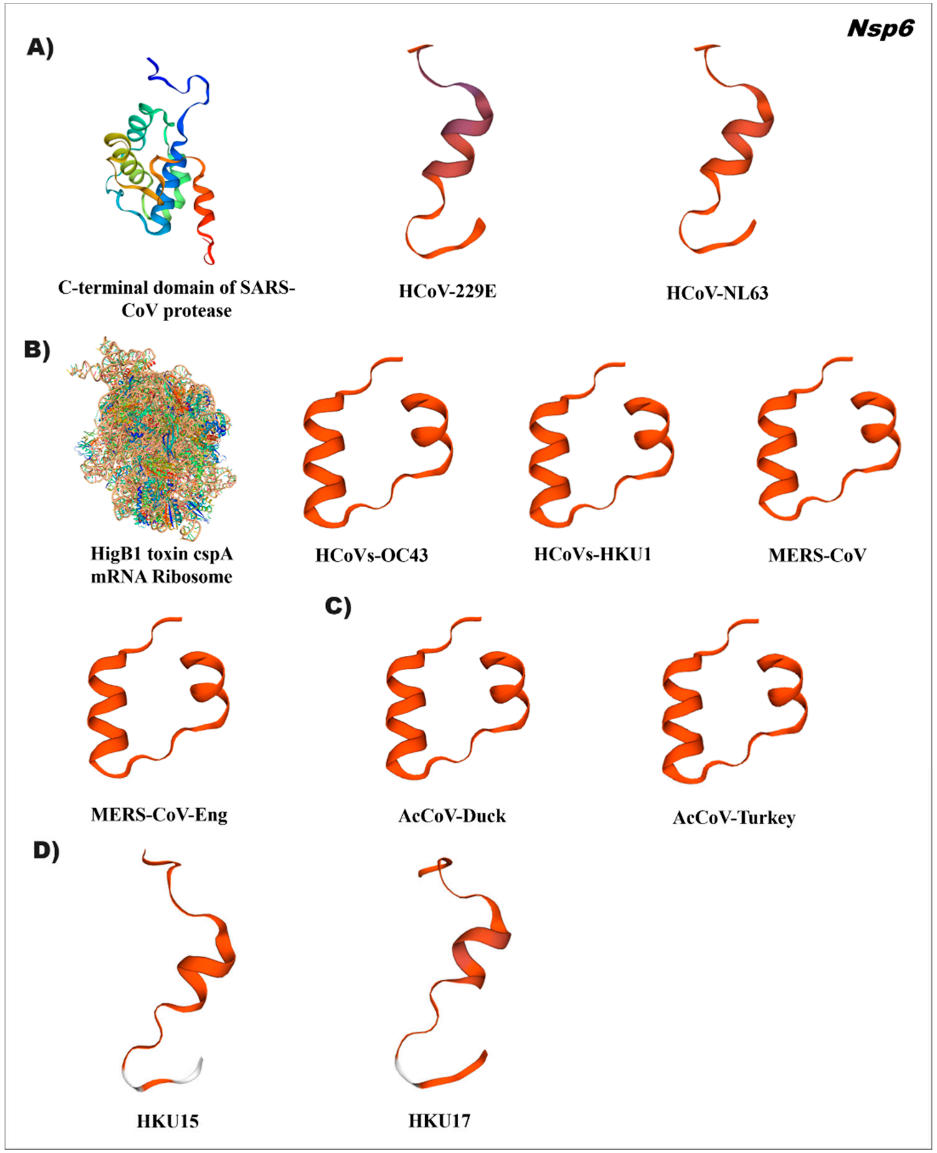
Nsp6
A)
C-terminal domain of SARS-CoV protease
HCoV-229E
HCoV-NL63
B)
HigB1 toxin cspA mRNA Ribosome
HCoVs-OC43
HCoVs-HKU1
MERS-CoV
C)
MERS-CoV-Eng
AcCoV-Duck
AcCoV-Turkey
D)
HKU15
HKU17

Supplementary Figure 1. Tertiary structure in silico of partial C-terminal of the Nsp6 of the coronavirus genus alpha, beta, gamma and delta. The prediction of the tertiary conformation was made using the SWISSMODEL program and the ID corresponding to each virus genus was used as a reference. **A)** Alphacoronavirus: HCoVs-229E (NP_073549.1), HCoVs-NL63 (YP_003766.2); **B)** Betacoronavirus: HCoVs-OC43 (YP_009555238.1), HCoVs-HKU1 (YP_173236.1), MERS-CoV (YP_009047202.1), MERS-CoV-Eng (YP_007188577.3); **C)** Gammacoronavirus: AcCoV-Duck (YP_009825007.1), AcCoV-Turkey (YP_001941164.2); **D)** Deltacoronavirus: Porcine coronavirus HKU15 (QWE80491.1), Sparrow deltacoronavirus HKU17 (AWV67106.1). Reference ID: 2K7x.1 (Alphacoronavirus and Deltacoronavirus), 7nbu.1 (Betacoronavirus and Gammacoronavirus). Tertiary structures were made using SWISS MODEL software.
